# Reservoir Dynamic Interpretability for Time Series Prediction: A Permutation Entropy View

**DOI:** 10.3390/e24121709

**Published:** 2022-11-23

**Authors:** Xiaochuan Sun, Mingxiang Hao, Yutong Wang, Yu Wang, Zhigang Li, Yingqi Li

**Affiliations:** 1 College of Artificial Intelligence, North China University of Science and Technology, Bohai Road, Tangshan 063210, China; 2 Hebei Key Laboratory of Industrial Perception, Tangshan 063210, China; 3 School of Computer and Communication Engineering, University of Science and Technology Beijing, Beijing 100083, China

**Keywords:** echo state network, time series prediction, interpretability, PE, reservoir richness, projection capability

## Abstract

An echo state network (ESN) is an efficient recurrent neural network (RNN) that is widely used in time series prediction tasks due to its simplicity and low training cost. However, the “black-box” nature of reservoirs hinders the development of ESN. Although a large number of studies have concentrated on reservoir interpretability, the perspective of reservoir modeling is relatively single, and the relationship between reservoir richness and reservoir projection capacity has not been effectively established. To tackle this problem, a novel reservoir interpretability framework based on permutation entropy (PE) theory is proposed in this paper. In structure, this framework consists of reservoir state extraction, PE modeling, and PE analysis. Based on these, the instantaneous reservoir states and neuronal time-varying states are extracted, which are followed by phase space reconstruction, sorting, and entropy calculation. Firstly, the obtained instantaneous state entropy (ISE) and global state entropy (GSE) can measure reservoir richness for interpreting good reservoir projection capacity. On the other hand, the multiscale complexity–entropy analysis of global and neuron-level reservoir states is performed to reveal more detailed dynamics. Finally, the relationships between ESN performance and reservoir dynamic are investigated via Pearson correlation, considering different prediction steps and time scales. Experimental evaluations on several benchmarks and real-world datasets demonstrate the effectiveness and superiority of the proposed reservoir interpretability framework.

## 1. Introduction

Reservoir computing (RC) [1] is widely recognized as a computational model suited for sequential data modeling. Its key component is the reservoir with a large number of sparsely and randomly connected neurons, capturing high-dimensional dynamic features of input data. Such an RC paradigm can avoid some drawbacks of gradient-descent RNN training, especially the time-consuming problem. The echo state network (ESN) is a popular RC model with low computational cost and powerful nonlinear projection capability [2]. Specially, the ESN training is fairly simple, since only the output weight is the training part, which is achieved by typical regression methods. Given these advantages, ESNs have been widely applied in the fields of time series prediction [3,4,5], image processing [6,7], feature extraction [8,9] and text classification [10]. Despite this fact, the ESN remains a black box due to its uninterpretable reservoir operation mechanism, i.e., high-dimensional projection [11,12]. It makes ESN hard to be a well-received paradigm for practical applications.

Currently, many efforts have been devoted to the RC interpretability for revealing its internal mechanism. Bianchi et al. pioneered the investigation of ESN interpretability by analyzing reservoir neuron dynamics via recurrence plots (RP) [13]. Such RP could determine the stability of the network, and the metric analysis of RP helped adjust important hyperparameters to push the ESN into the stability boundary. Moreover, RP theory was also used to explain the self-organizing convolutional echo state network proposed by Lee et al. [14]. Bianchi et al. adopted a horizontal visibility graph approach to reflect ESN dynamics, which guided the adjustment of hyperparameters [15]. Ceni et al. suggested an excitable network attractor method to explain the operational mechanism of ESNs in specific tasks [16]. Variengien et al. proposed a recurrent state space visualization method, visualizing the learning process of ESNs, as well as revealing the effects of hyperparameters on reservoir dynamics [17]. Armentia et al. tried to illustrate how perturbed features affected the readout of ESNs using a perturbation-based importance attribution method [18]. Arrieta et al. presented a set of Explainable Artificial Intelligence techniques to visualize the potential memory, temporal patterns, and pixel absence effect of the model, thereby enabling the interpretation of DeepESN [19]. In the task of analyzing dynamic systems using ESN, Alao et al. interpreted the learned reservoir output weights as a representation of system dynamics through principal component analysis [20]. Baptista et al. used the SHapley Assitive exPlanations method for ESN to reveal the effect of different input features on the prediction results [21]. Some attempts devote to designing reservoir structures with high interpretability. Han et al. introduced an interpretable directed acyclic network for RC, where the effects of reservoir neurons on prediction performance were characterized by analyzing the memory property of each neuron [22]. Gauthier et al. designed an interpretable implicit RC model based on nonlinear vector autoregression to solve the reservoir uncertainty problem, i.e., random generation and multiple hyperparameters [1]. Miao et al. designed adaptive reservoirs based on the implementation theory of linear dynamical systems for a given task [23]. Despite these studies, for the reservoir with a large number of randomly sparsely connected neurons, it remains elusive how this uncertain structure can yield excellent performance. In other words, the correlation between reservoir richness and its high-dimensional projection capability remains unclear.

The permutation entropy (PE) [24] can reveal the complexity of time series by means of subsequence sorting in a high-dimensional reconstruction space [25]. The method is computationally simple, noise-resistant, and sensitive to local variations, thus possessing the powerful capacities of mutation information identification and visualization [26]. PE has been widely employed in fault detection [27], electrocardiography [28], complex systems [29], and other domains. To open the door for a wider acceptance of the ESN methodology, we explore the feasibility of the PE approach to explain good reservoir projection capability. The detailed motivation for the research in this paper can be found in the Section 2.1.

In this article, we tackle the interpretability issue of an ESN by analyzing the instantaneous and global dynamics of reservoir neurons with the PE measure. Such a method can quantify the information of the states in a high-dimensional dynamical systems over time. We believe that the PE analysis is a valuable tool for a deep insight of dynamic reservoir behavior. The contributions of this paper can be summarized as follows.

- We develop two reservoir dynamic analysis methods based on PE from the instantaneous and global modeling perspectives. This is the first attempt to use PE and its multiscale modeling tools to reveal the relationship between reservoir richness and projection capacity.
- We investigate the sensitivity of ISE and GSE on hyperparameters affecting reservoir richness.
- We use multiscale complexity–entropy to analyze the global reservoir and neuron-level states to verify the single-scale and input-driven properties of reservoirs.
- We reveal the multistep and multiscale relationships between ESN performance and reservoir dynamic, which is achieved by measuring the Pearson correlations between nonlinear approximation/memory capacity and global PE of neurons’ states.

This paper is constructed as follows. In Section 2, we provide an introduction to the preparatory knowledge, which includes research motivation and ESN architecture. In Section 3, we provide the permutation entropy method and explain how reservoir dynamics can be investigated by employing PE. In Section 4, experiments are completed utilizing the PE approach, and the experimental data are examined for their insight into reservoir dynamics. In Section 5, we provide a detailed discussion on the relationship between ESN performance and reservoir state entropy. Finally, Section 6 presents final observations and future study directions.

## 2. Preliminary

### 2.1. Motivation

Since the ESN was proposed, a rich body of existing work has driven the progress of ESNs. Due to the ill-posed problem with the original ESN, much research has focused on topology construction, training methods, or hyperparameters tuning. However, the uninterpretable reservoir can greatly limit the application and advancement of ESN. Like traditional RNN, the reservoir states of ESN are continuously updated with time. Considering the temporal state of each neuron as a one-dimensional state, the whole reservoir state is regarded as a high-dimensional time-variant system. For this input-driven system, the reservoir layer neuron states are updated under the guidance of a randomly generated matrix *W*. Why is such a state update mode conducive to competitive reservoir projection and even high-efficient (remarkable) nonlinear approximation capacity? It is worth further study; thus, the focus of this paper is to explore the feasibility of revealing the good performance of ESN from the perspective of reservoir state analysis.

As stated in Section 1, this paper intends to focus on PE-based reservoir interpretability. In fact, Ozturk et al. defined the average state entropy (ASE) using Renyi’s quadratic entropy and first proposed the concept of reservoir richness [30]. Furthermore, Gallicchio et al. used ASE as an indicator of reservoir complexity in DeepESN to support the applicability of multi-reservoir models [31]. However, both of them did not clarify the relationship between reservoir richness and reservoir projection capacity, and the significance of increasing reservoir richness is not discussed. On the other hand, ASE has been proposed only from the perspective of instantaneous reservoir states, neglecting more detailed neuronal time-varying states. Although Renyi’s entropy can measure the sequence complexity, it is slightly inferior to PE in detecting dynamic mutations because it does not consider the ordering pattern of the system. Given these discussions, the motivation of this paper is to model the reservoir dynamics from multiple perspectives based on PE to reveal the correlation between reservoir richness and nonlinear projection capacity.

### 2.2. Esn Architecture

ESN is a popular RNN paradigm with a special hidden layer called a reservoir, which is generated from randomly and sparsely connected neurons. Such an information processing unit can effectively achieve the high-dimensional feature mapping of input data.

**Figure 1:**
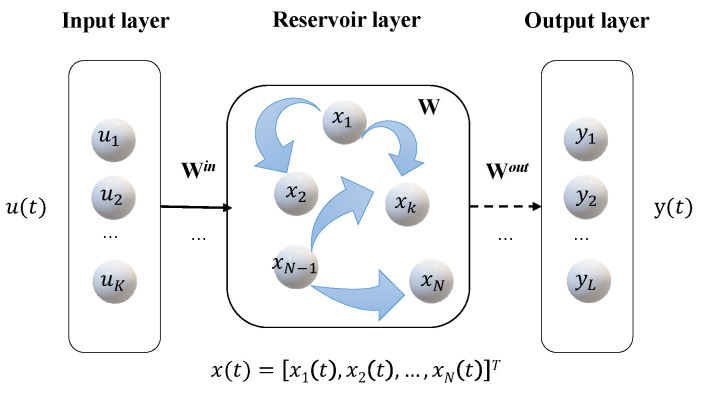
A sketch of the ESN architecture, where the output feedback of the reservoir is not considered.

Figure 1 depicts the ESN structure. Obviously, ESN is composed of three layers: an input layer with *K* input units, a reservoir with *N* internal units, and an output layer with *L* output units. There exist the following types of connections, each of which has its own weight matrix, i.e., the input weight matrix $W^{in}\in R^{N\times K}$ for the weights from input units to internal ones, the internal weight matrix $W\in R^{N\times N}$ for the weights between internal units, and the output weight matrix $W^{out}\in R^{L\times (N+K)}$ for the weights from input and internal units to output ones. Generally, $W^{in}$ and *W* are generated randomly and fixed during the ESN training. Only $W^{out}$ (dashed line in Figure 1) is the trained part.

For a given input signal $u\left(t\right)={\left[u_{1}\left(t\right),u_{2}\left(t\right),\ldots ,u_{K}\left(t\right)\right]}^{T}$, the state of the driven reservoir at time step *t* can be expressed as

(1) $$x\left(t\right)=f(W^{in}u\left(t\right)+Wx\left(t-1\right)),$$

where f(·) is the activation function of internal units (sigmoid in our consideration). The corresponding network readout of this ESN is given by

(2) $$y\left(t\right)=W^{out}x\left(t\right).$$

Once the internal state *X* and the desired output *Y* are collected, $W^{out}$ can be effortlessly obtained by solving the following least squares problem

(3) $$\underset{W^{out}}{min}\left|\right|W^{out}X-Y{\left|\right|}_{2}^{2}$$

and its closed-form solution is calculated by a ridge regression in our scenario, that is

(4) $$W^{out}=Y\cdot X^{-1}$$

It means the completion of the ESN training.

## 3. Methodologies

Here, we speak about how PE-based methodologies can be utilized to analyze the input-driven dynamics of an ESN reservoir. In Section 3.1, we discuss the PE algorithm in detail and describe our analysis method using the reservoir state as the analysis object; in Section 3.2, we discuss multiscale permutation entropy and statistical complexity measure (SCM) and how to use them to analyze the reservoir state of an ESN.

### 3.1. Permutation Entropy Measure of Reservoir Dynamics

**Figure 2:**
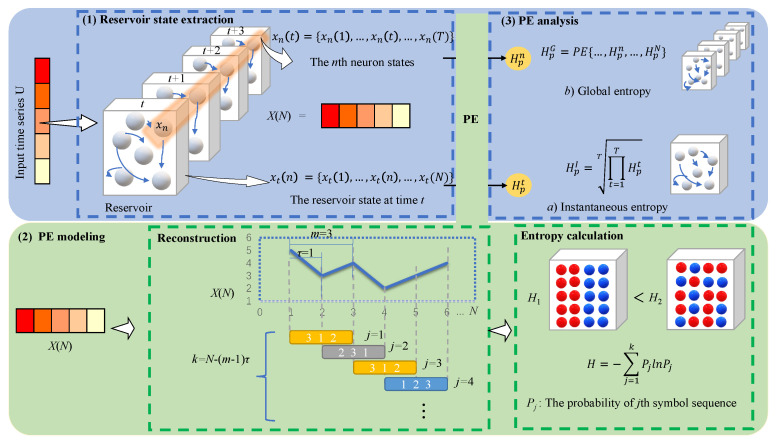
PE-based Interpretability framework for RC.

Figure 2 gives an overview of our PE-enabled reservoir state analysis framework. In structure, this framework is composed of the following three functionalities, i.e., the extraction, PE modeling and PE analysis of reservoir states. First, two types of reservoir states are extracted during training, namely the instantaneous state of all reservoir neurons at certain moment and the individual neuron state over time. Then, the PE modeling is performed for entropy calculations of the two different reservoir state sequences extracted. Afterwards, the entropy analysis is used to quantitatively estimate the instantaneous and global reservoir entropies in order to measure the degree of reservoir richness.

In the following, we elaborate the PE modeling of reservoir states, including phase space reconstruction, sorting, entropy calculation. Assuming the reservoir state $x_{t}\left(n\right)=\left\{x_{t}\left(1\right),\ldots ,x_{t}\left(n\right),\ldots ,x_{t}\left(N\right)\right\}$ at time *t*, we can obtain a reconstructed phase space with k=N−(m−1)τ components, which is described as follows

(5) $$J=\left[\begin{array}{cccc} x_{t}\left(1\right) & x_{t}\left(1+\tau \right) & \ldots & x_{t}\left(1+\left(m-1\right)\tau \right)\\ \ldots & \ldots & \; & \ldots \\ x_{t}\left(j\right) & x_{t}\left(j+\tau \right) & \ldots & x_{t}\left(j+\left(m-1\right)\tau \right)\\ \ldots & \ldots & \; & \ldots \\ x_{t}\left(k\right) & x_{t}\left(k+\tau \right) & \ldots & x_{t}\left(k+\left(m-1\right)\tau \right) \end{array}\right],$$

where *N* is the number of reservoir neurons, while *m* and τ denote the embedding dimension and the delay time, respectively. Considering the *j*th reconstructed component J(j), the *m* data in this component are rearranged in the order of smallest to largest. Specially, if any two elements of J(j) are equal, their orders are not changed. Consequently, the sorted data can be expressed as follows

(6) $$X_{j}=x_{t}\left(j+\left(l_{1}-1\right)\tau \right),x_{t}\left(j+\left(l_{2}-1\right)\tau \right),\cdots ,x_{t}\left(j+\left(l_{m}-1\right)\tau \right),$$

where $L=\{l_{1},l_{2},\ldots ,l_{m}\}$ denote the column index of each element of the phase space after sorting. The *m*-dimensional phase space has the possible permutations up to m!. Using the Shannon entropy calculation method and normalizing, we can obtain the state entropy $H_{p}^{t}$ of all neurons in the reservoir at time *t*. Furthermore, for the *n*th neuron with reservoir state $x_{n}\left(t\right)=\left\{x_{n}\left(1\right),\ldots ,x_{n}\left(t\right),\ldots ,x_{n}\left(T\right)\right\}$, the time-varying state entropy $H_{p}^{n}$ of a given neuron can be found by the above method. The formula for calculating $H_{p}^{t}$ and $H_{p}^{n}$ is given as

(7) $$\left\{H_{p}^{t},H_{p}^{n}\right\}=-\frac{\sum_{j=1}^{k}P_{j}lnPj}{ln(m!)},$$

where $P_{1},P_{2},\ldots ,P_{k}$ denote the occurring probability of each sequence. Obviously, the entropy calculations of these two different reservoir states follow the same expression.

Finally, our PE analysis focuses on the ISE and the GSE of the reservoir. Based on Equation (7), ISE can be obtained by the geometric mean of all $H_{p}^{t}$ over all moments, which is denoted as follows

(8) $$H_{p}^{I}=\sqrt[{T}]{\prod_{t=1}^{T}H_{p}^{t}},$$

where *T* denotes the entire training time. The reservoir GSE is calculated by solving the normalized Shannon entropy of the sequence $\left\{H_{p}^{1},\ldots ,H_{p}^{n},\ldots ,H_{p}^{N}\right\}$ consisting of the time-varying state entropies of all reservoir neurons, which are given by

(9) $$H_{p}^{G}=PE\left\{H_{p}^{1},\ldots ,H_{p}^{n},\ldots ,H_{p}^{N}\right\}.$$

In our consideration, ISE aims at reflecting the nonlinear projection capability of the reservoir by the measure of instantaneous reservoir richness. GSE considers the neuron level on the basis of ISE and reveals different factors affecting reservoir richness. The detail of these two reservoir dynamic analysis can be found in Algorithm 1.
**Algorithm 1** PE analysis on reservoir states**Input:** Datasets**Output:**$H_{p}^{I}$, $H_{p}^{G}$ 1:Initialize ESN 2:**for** n→N**do** 3:    **for** t→T **do** 4:        Collect reservoir states 5:    **end for** 6:**end for** 7:Obtain the ESN state matrix *X* 8:Extract the reservoir state: $x_{t}\left(n\right)$, $x_{n}\left(t\right)$ 9:Initialize PE parameters *m*, τ 10:**for** $x_{t}\left(n\right)$, $x_{n}\left(t\right)$**do** 11:    Obtain $J_{km}$ based on Equation (5) 12:    **for** j→k **do** 13:        Obtain $X_{j}$ based on sorting to J(j) 14:        Obtain the sequence of symbols *L* 15:    **end for** 16:    Calculate the probability *P* of occurrence of *L* 17:    Calculate $H_{p}^{t}$ and $H_{p}^{n}$ based on Equation (7) 18:**end for** 19:Obtain ISE $H_{p}^{I}$ and GSE $H_{p}^{G}$ based on Equations (8) and (9) 20:**return**$H_{p}^{I}$, $H_{p}^{G}$

### 3.2. Multiscale Complexity-Entropy Analysis

To further explore the reservoir dynamics, here, we use a multiscale complex permutation entropy (MCPE) method. The multiscale permutation entropy and statistical complexity are described below.

Multiscale permutation entropy (MPE) is an improvement PE. The basic idea is to first multiscale coarse-grain the time series and then calculate its PE. For a reservoir state $x_{t}=\left\{x_{t}\left(1\right),\ldots ,x_{t}\left(n\right),\ldots ,x_{t}\left(N\right)\right\}$ at time *t*, the versions $x_{j}^{s}$ at different scales can be obtained by the following coarse-grained decomposition:

(10) $$x_{j}^{s}=\frac{1}{s}\sum_{i=(j-1)s+1}^{js}x_{i},$$

where *s* is the scale factor, and *j* is the sequence index after coarse granulation, following $1\leq j\leq \frac{N}{s}$. Specially, when s=1, the MPE is the traditional PE. After the above decomposition, using Equations (7) and (10), we can obtain the GSE $H_{p}^{G}$ and the neuron-level $H_{p}^{n}$ at different scales, respectively, i.e., the MPE.

Furthermore, we give the SCM of reservoir dynamics, which is derived from the product of the entropy and the disequilibrium. This measure is able to grasp the essential details of dynamics while discerning different degrees of periodicity and chaos. Given a probabiliby distribution *P* in Equation (7), the SCM can be defined by the product of the normalized permutation entropy $H_{p}$ and a suitable metric distance $Q_{j}\left[P,P_{e}\right]$, which is expressed as follows:

(11) $$C_{js}=Q_{j}\left[P,P_{e}\right]H_{p}.$$

In this formula, $H_{p}$ refers to the $H_{p}^{G}$ in GSE perspective, or the $H_{p}^{n}$ at the neuron-level, therefore, $C_{js}$ stands for the global reservoir complexity or single neuron complexity, respectively, and $Q_{j}\left[P,P_{e}\right]$ represents the degree of difference of the probability $P=\{P_{1},\ldots ,P_{k}\}$ from the uniform distribution $P_{e}=\left\{P_{e_{1}},\ldots ,P_{e_{k}}\right\}=\left\{\frac{1}{m!},\ldots ,\frac{1}{m!}\right\}$, which is given by

(12) $$Q_{j}\left[P,P_{e}\right]=\frac{S\left[\left(P+P_{e}\right)/2\right]-S\left[P\right]/2-S\left[P_{e}\right]/2}{Q_{max}},$$

where *S* denotes the un-normalized information entropy, and $Q_{max}$ is the maximum possible value of $Q_{j}\left[P,P_{e}\right]$, which is calculated as follows:

(13) $$Q_{max}=-\frac{1}{2}\left[\frac{m!+1}{m!}\log\left(m!+1\right)-2\log\left(2m!\right)+\log\left(m!\right)\right],$$

where m! is all possible permutations mentioned in Section 3.1.

The combination of the above MPE and SCM measures form our MCPE approach for interpreting reservoir dynamics. On the one hand, the MCPE is able to demonstrate time series complexity trends at multiple scales. In our consideration, it is used to detect reservoir multi-scale dynamics [32]. On the other hand, it can distinguish random and chaotic behaviors through delineating the representation space known as the complexity entropy causal plane (CECP) [33].

## 4. Experiments

In this section, the corresponding experiment evaluation is conducted to verify the superiority of the proposed interpretability method. We consider four different datasets, including three classical benchmark tasks for time series modeling and a real-world dataset. First, we devote to revealing its remarkable nonlinear projection from the views of instantaneous and global reservoir dynamic in the PE analysis framework. It can be achieved by the impact analysis of reservoir hyperparameters on its state entropy experimentally. On the other hand, such reservoir dynamics is further dissected more deeply by a multiscale approach.

In the following experiments, we consider an ESN with no output feedback, where $W^{in}$ and *W* are randomly generated in the interval [−1,1], the connectivity of *W* is 0.05, and $W^{out}$ is trained by the ridge regression with the regularization factor of $10^{-6}$. To highlight the performance comparison, we add the noise obeying a Gaussian distribution with the variance of 0.1 for the used four datasets. During ESN training, according to the standard dropout procedure, the first 100 elements of the training data are discarded to remove the transients of the ESN. Specially, we use a fixed invariant random number seed in all experiments, and the permutation entropy is computed by the ordpy Python library proposed by Pessa et al. [34].

### 4.1. Dataset

**Figure 3:**
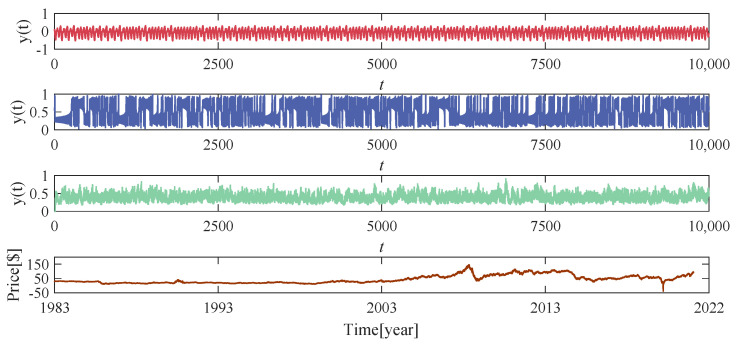
A sample of data. From top to bottom are the MG system, the Lorenz system, the NARMA system, and the CO for real-time timing data. The horizontal coordinate indicates the time step, a total of 10,000.

Three benchmark time series and one real-world dataset are considered, including the chaotic mapping system Mackey–Glass (MG) and Lorenz, the stochastic model nonlinear autoregressive moving average (NARMA), and the New York crude oil market average price (CO). Figure 3 shows the trends of these time series over time.

As is known, ESN is preferred in dealing with chaotic time series by virtue of its outstanding nonlinear projection capability on MG and Lorenz systems. The MG system is generated by the following equation:

(14) $$\frac{dy(t)}{dt}=\frac{0.2y(t-\tau )}{1+y^{10}\left(t-\tau \right)}-0.1y\left(t\right),$$

where the whole sequence is chaotic, acyclic, and non-divergent (convergent) when τ>17, and 10,000 sample points are used at τ=17. The Lorenz chaotic attractor [35] is defined as follows

(15) $$\begin{array}{c} \dot{x}=\sigma \left(y-x\right)\\ \dot{y}=x\left(\rho -z\right)-y\\ \dot{z}=xy-\beta z+x, \end{array}$$

In this formula, σ=10, β=8/3, ρ=28 in general.

As a typical discrete-time system, NARMA has been widely utilized for the performance assessment of neural networks. Effectively modeling NARMA is a difficult task due to its nonlinearity and long-memory problems. In our experiments, the tenth-order NARMA system is considered, which is generated by

(16) $$y\left(t+1\right)=0.3y\left(t\right)+0.05y\left(t\right)[\sum_{i=0}^{9}y\left(t-i\right)]+1.5u\left(t-9\right)u\left(t\right)+0.1,$$

where u(t) and y(t) denote the input and output at moment *t*, respectively, which are randomly yielded from a uniform distribution with the interval [0,1].

Finally, the real-world dataset on the daily closing spot price of the West Texas Intermediate is used for our experiment evaluation. It can be collected from the US Energy Information Administration website over the time range from 4 April 1983 to 23 February 2022 (N=9766 oil price observations), which is quoted in US dollars per barrel. As proved in [36], oil price time series have strong stochastic properties; thus, its accurate prediction is exceedingly challenging.

### 4.2. ISE Analysis

**Figure 4:**
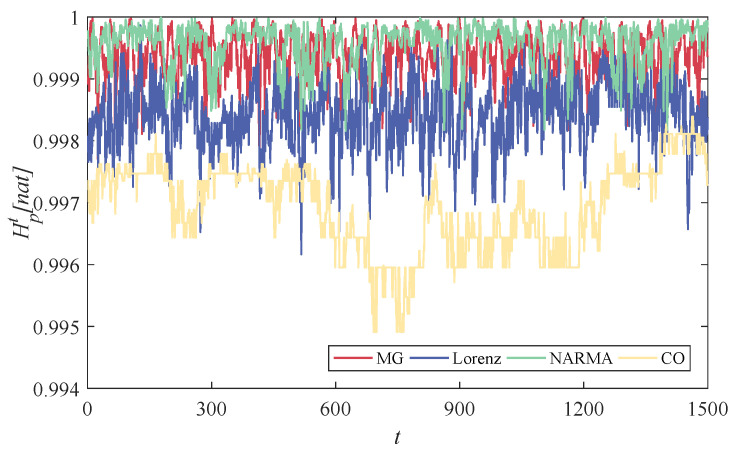
PE of the normalized reservoir states over time for the considered four time series prediction tasks, where the horizontal coordinate denotes the whole state updating steps, and the vertical coordinate denotes the corresponding PEs.

In Figure 4, we plot the state entropy of all reservoir neurons at each moment for different time series prediction tasks, i.e., $H_{p}^{t}$. From this figure, it is obvious that these normalized entropy values fluctuate in the range between 0.995 and 1. Such a high PE over all moments reflects the considerable complexity of different neuron states, indicating significant differences among these reservoir neurons [37]. It can effectively alleviate the collinearity issue of the reservoir, thus obtaining an excellent nonlinear projection capability [38]. On the other hand, the state entropy of the reservoir always fluctuates steadily within a specific range, which means that the reservoir retains high richness over time, even though each neuron’s state continuously updates. In addition, the entropy of reservoir states is task dependent, interpreting the input-driven mechanism of ESNs.

**Figure 5:**
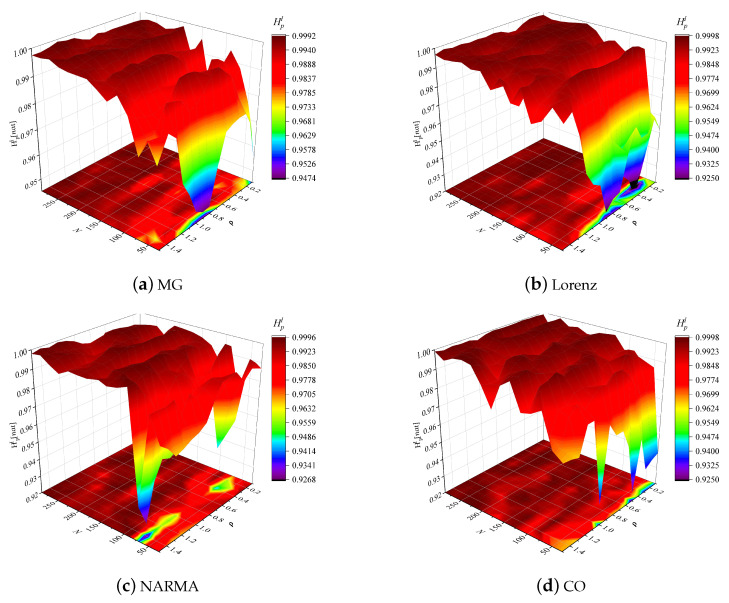
Reservoir ISEs versus reservoir size *N* and spectral radius ρ in the MG, Lorenz, NARMA and CO prediction tasks.

To explicitly illustrate the effects of hyperparameter tuning on reservoir richness, we visualize the ISE variation against spectral radius ρ and reservoir size *N* applied to the MG, lorenz, NARMA and CO datasets, as shown in Figure 5. From this figure, there exists the significant fluctuation of $H_{p}^{I}$ as *N* decreases, meaning that the high entropy in low-size reservoirs hardly occurs. For the small *N*, the reservoir dynamic appears to be more sensitive to spectral radius. Intuitively, ρ∈[0.6,1] seems to be the best choice in the modeling of NARMA and CO datasets, while the relatively small or large ρ should be considered for MG and Lorenz, respectively, i.e., ρ∈[0.2,0.7] or ρ>1. In the contrary, the large-scale reservoir (N≥150) is more conducive to yielding high ISE, and in this case, ρ has a slight effect on the ISE. Such a finding can be illustrated by the fact that a large reservoir size can determine the richness of a randomly connected reservoir. While the spectral radius is more suitable for ensuring the echo state and stable edges of the ESN, the effect on richness is uncertain [39].

### 4.3. GSE Analysis

**Figure 6:**
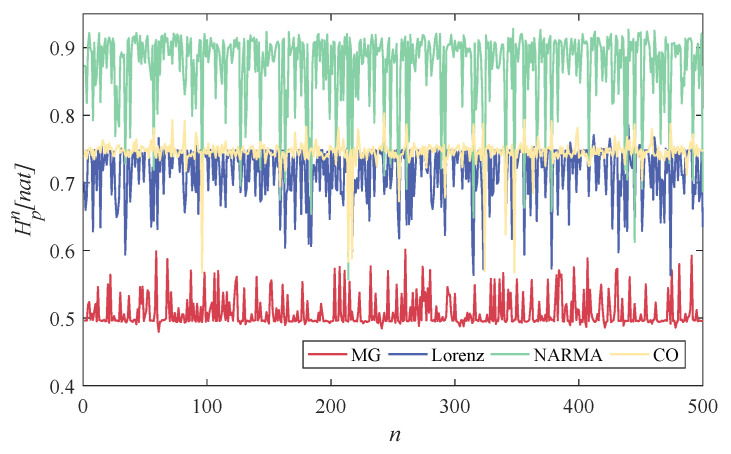
PEs of time-varying states of different reservoir neurons for the considered four time series prediction tasks, where the horizontal coordinate denotes each neuron in the reservoir, and the vertical coordinate denotes the corresponding PEs.

As is known, the states of reservoir neurons are continuously updated during the training process. The data are gradually uploaded into the reservoir as time flows, and each neuron thus obtains a time-varying state sequence. Investigating the neuron-level time-varying effect can provide an insight of neuron-level projection. Figure 6 depicts the time-varying state entropy of each reservoir neuron for different time series prediction tasks, i.e., $H_{p}^{n}$. Obviously, from a global view, such neuron-level entropy is task dependent, where it fluctuates around consistently high levels for the CO and NARMA series rather than the MG series.

On the one hand, from the task view, the difference in the low and high entropy values is mainly caused by the different complexity of the input series, where the complexity is measured by the PE method, as listed in Table 1, and the bigger PE means the more significant complexity. On the other hand, for the same prediction task, the neurons generated from the randomly generated and sparsely connected reservoir have significant differences of entropy, reflecting different activation levels of the reservoir neurons [40]. This interindividual variability between neurons provides a support for our investigation of reservoir richness.

**Figure 7:**
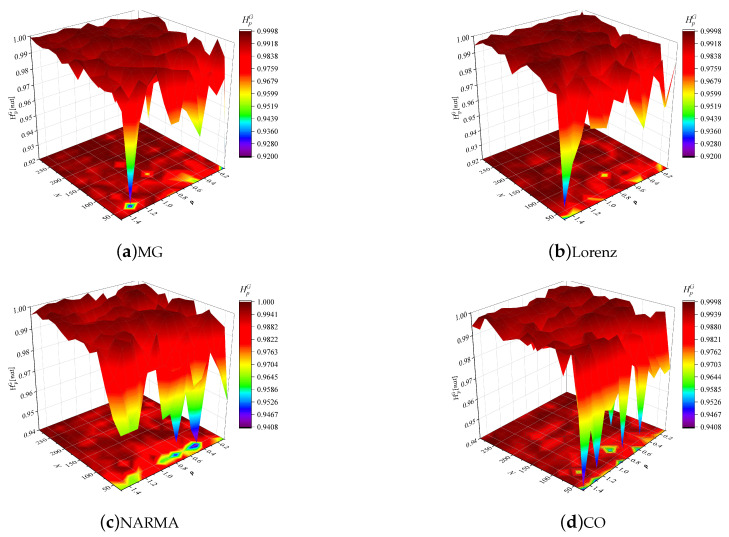
Reservoir GSEs versus reservoir size *N* and spectral radius ρ in the MG, Lorenz, NARMA and CO prediction tasks.

**Figure 8:**
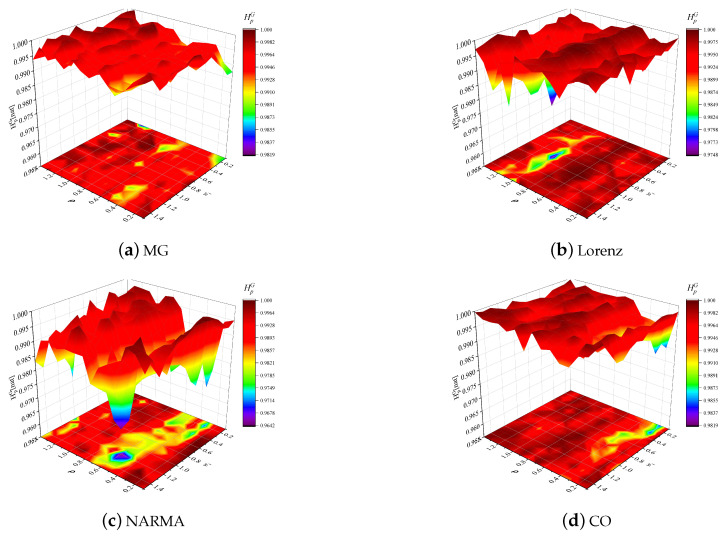
Reservoir GSEs versus reservoir size *N* and spectral radius ρ in the MG, Lorenz, NARMA and CO prediction tasks.

Figure 7 evaluates the effects of *N* and ρ on the reservoir GSEs in different time series prediction tasks, where this global entropy is obtained by calculating the entropy of the time-varying state entropies of all reservoir neurons in Figure 6. Apparently, similar to the ISE visualization, the joint tuning of these two hyperparameters can enable the ESN to obtain an optimal GSE for these prediction tasks, guaranteeing reservoir richness, and the reservoir size is still the most influential hyperparameter for reservoir dynamic. Furthermore, we also evaluate the effects of input scaling $\omega_{i}$ and spectral radius ρ on the GSE in the case of N=200, as shown in Figure 8. Obviously, the reservoir GSE always fluctuates at the level of high values, and ρ has a more significant effect on the GSE than $\omega_{i}$, determining the reservoir projection capability more sensitively.

### 4.4. Multiscale Reservoir Dynamics

**Figure 9:**
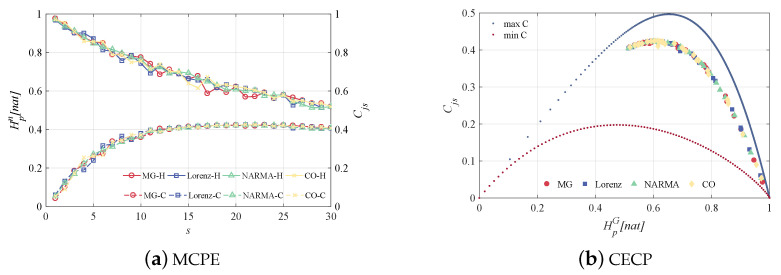
Multiscale global reservoir dynamics in the MG, Lorenz, NARMA and CO prediction tasks. (**a**) MCPE, where the solid and dashed lines are the variation curves of entropy and complexity, respectively, (**b**) CECP, where the top and bottom scatters represent the maximum and minimum complexity.

Here, we concentrate on the MCPE experiments for global reservoir dynamic and its neuron-level dynamic, respectively, allowing the dissection of complex systems at multiple scales and helping characterize reservoirs more effectively. Figure 9 shows the GSE of reservoirs at different scales for the considered time-series prediction tasks as well as the relationship between complexity and GSE, i.e., the complexity–entropy causality plane (CECP). From the MCPE plot, it is observed that as the scale *s* increases, the GSE decreases continuously, while the statistical complexity $C_{js}$ increases, and consequently, their changes tend to level off. It is noticeable that from our coarse-grained treatment, the reservoir has the highest GSE at the single scale, i.e., s=1, and the larger *s* can yield a less complex reservoir. Such a single-scale phenomenon illustrates the significant difference between reservoir neuron states, meaning weak collinearity. Hence, the reservoir has a strong nonlinear projection capability. In Figure 9b, larger entropy values correspond to smaller $C_{js}$, which is a piece of strong evidence for reservoir stochasticity. The effect of different prediction tasks on reservoir richness is less obvious here.

**Figure 10:**
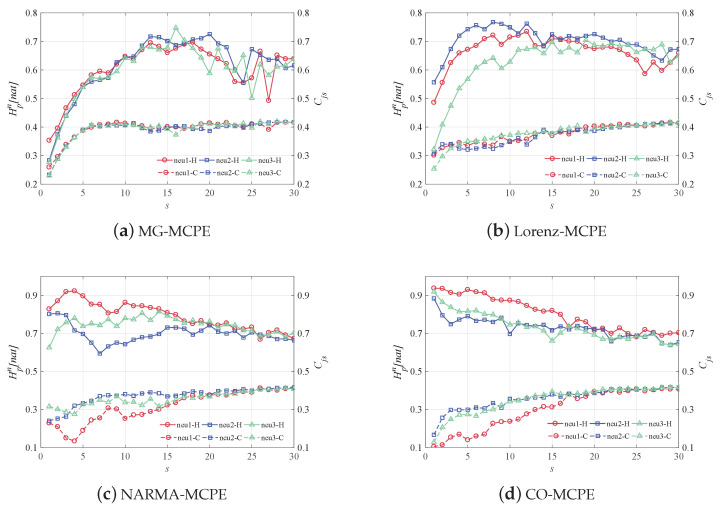
The MCPEs of three randomly selected neurons for the MG, Lorenz, NARMA, and CO datasets, where the solid line indicates the entropy change curve of $H_{p}^{n}$, and the dashed line indicates the complexity change curve of $C_{js}$.

**Figure 11:**
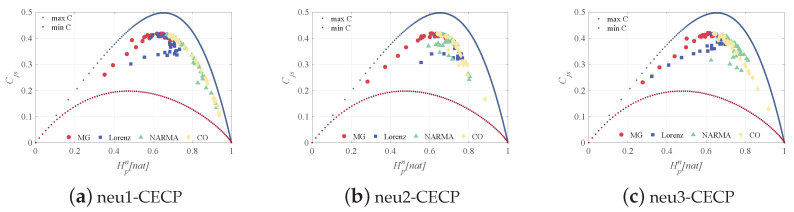
The CECPs of three randomly selected neurons for the MG, Lorenz, NARMA, and CO datasets, where the top and bottom lines represent the maximum and minimum complexity, respectively, and the different symbolic markers are used to distinguish the datasets.

From a neuron-level view, Figure 10 depicts the multiscale dynamic metric of the time-varying state of a single reservoir neuron for different time series prediction tasks. For four data-driven reservoirs, we consider the same three neurons to reveal their time-varying state features. For the MG prediction task in Figure 10a, as *s* increases, $H_{p}^{n}$ increases sharply and then decreases slowly, and $C_{js}$ increases and then tends to be stable. The $H_{p}^{n}$ of Lorenz-driven reservoir neurons increased rapidly at low scales, peaked at s=7 and then leveled off. Compared with Figure 10a, the neuronal entropy values under the Lorenz system appear significantly different at low scales. For the NARMA and CO tasks, as *s* increases, $H_{p}^{n}$ decreases and $C_{js}$ slowly increases, as shown in Figure 10c,d. It is worth noting that the evident differences between neurons also appear at low scales. The above phenomenon demonstrates the difference in the activation level of neurons under different tasks. Because the time-varying states of neurons are driven by the inputs, the reservoir can effectively capture the input features, which ensures the ability of the reservoir to project nonlinearly for different tasks. In Figure 11, for the reservoir neuron of the MG task, the $H_{p}^{n}$ only reaches a maximum of 0.7 with increasing $C_{js}$, which means that the neuron exhibits the same chaotic nature as the MG dataset. A similar situation also occurs for the Lorenz task, since the yielded $H_{p}^{n}$ and $C_{js}$ are scattered in the central region of the CECP. In addition, the other two neuron time-varying state sequences have the same stochastic nature as the input data NARMA and CO. The reservoir neurons have the same properties as the original input data, further supporting the preservation of the input features on the reservoir and revealing the stability of the reservoir.

## 5. Discussion

In this section, to highlight the critical contributions, we give a detailed discussion on the relationships between the ESN performance and reservoir dynamic. The former refers to nonlinear approximation performance and memory capacity (MC), while the latter denotes the ISE and GSE of reservoirs. Specially, mean square error (MSE) is used for the approximation measure, which is given by

(17) $$MSE=\frac{1}{l_{test}}\sum_{t=1}^{l_{test}}{\left(y_{test}\left(t\right)-{\hat{y}}_{test}\left(t\right)\right)}^{2},$$

where $l_{test}$ is the test sample length, and $y_{test}\left(t\right)$ and ${\hat{y}}_{test}\left(t\right)$ are the test output and desired output at time *t*. The MC of ESN is defined as a reconstruction ability of the input from the past moment *t*, which is calculated as follows

(18) $$MC=\sum_{k=1}^{\infty }MC_{k}.$$

where *k* denotes the input delay (k=1,2,⋯,40 in our scenario), and $MC_{k}$ is actually the correlation coefficient between the input and the actual output by delaying *k* steps, which is given by

(19) $$MC_{k}=\frac{Cov^{2}\left(u\left(t-k\right),y\left(t\right)\right)}{Var(u(t))Var(y(t))},$$

where Cov denotes covariance, Var denotes variance, and u(t) and y(t) denote the input and output of the ESN at moment *t*, respectively.

**Figure 12:**
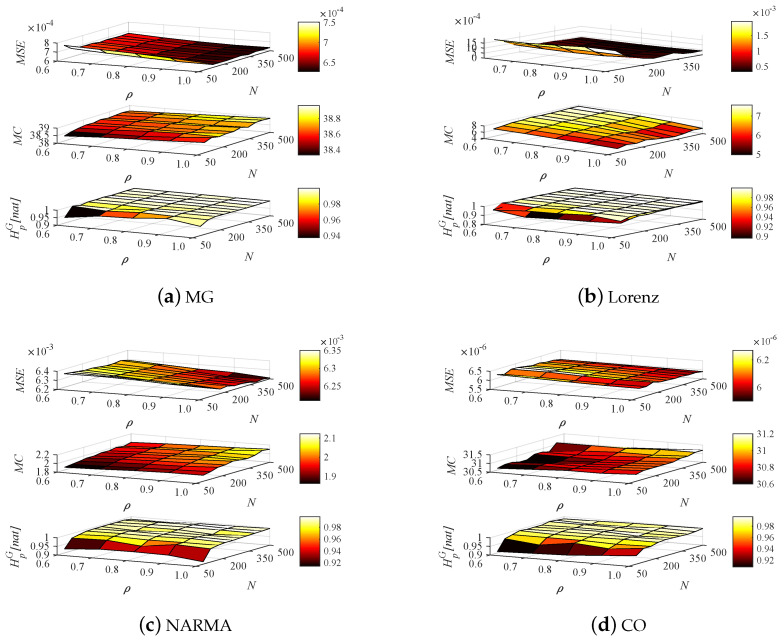
ESN performance versus reservoir size *N* and spectral radius ρ in the MG, Lorenz, NARMA and CO prediction tasks.

Figure 12 evaluates the effectiveness of ESN for the hyperparameters *N* and ρ in the considered time series prediction tasks. Obviously, there is a very good consistency between the ESN performance (MSE and MC) and the reservoir richness measured by $H_{p}^{G}$ via the whole parameter spaces of *N* and ρ. Concretely, the larger *N* can yield the lower MSE but the higher MC and $H_{p}^{G}$. Furthermore, Table 2 shows the measure between Pearson correlation $H_{p}^{G}$ and ESN performance for different time series tasks, especially considering multi-step ahead prediction mode. From the table, it is obvious that $H_{p}^{G}$ has an extremely strong correlation with the MSE and MC over the parameter space of *N*. Even if the prediction step is up to 5, such a high correlation still remains. It implies that in all settings of *N*, the reservoir with rich dynamics can be guaranteed, thus yielding good nonlinear projection capability. Given this, the PE theory is feasible for interpreting reservoir projection. On the other hand, from the view of spectral radius, the entropy–performance correlation is significantly weakened. It is due to the fact that the spectral radius just plays a decisive role in the convergence of reservoir internal weight matrix *W*, ensuring the echo state property, thereby having a slight effect on the richness of the reservoir.

Finally, Table 3 shows the MSEs and GSEs for different tasks as the scale increases. In this table, we can find that as the scale increases, the $H_{p}^{G}$ decreases while the prediction error of the model increases. This implies that the reservoir has a more powerful nonlinear projection capacity in the single-scale case. On the other hand, the different $H_{p}^{G}$ and MSE appear for the considered prediction tasks. These two comments are actually consistent with the single scale and input-driven findings from Figure 9, Figure 10 and Figure 11.

## 6. Conclusions

In this work, we gain insight into the excellent performance of RC from a PE interpretable framework. In particular, we define two metrics for reservoir analysis (ISE and GSE), which serve as a link between reservoir richness and reservoir projection capability. In addition, multiscale complexity–entropy tools are used to explore the dynamics of reservoir states and neuron-level states at different scales. The simulation results demonstrate the positive correlation between reservoir richness and reservoir projection capacity as well as the single-scale and input-driven properties of the reservoir. In future work, we would like to consider PE to analyze the richness of some deep reservoir structures (e.g., DESN) for the projection interpretability. Moreover, the mutual information derived from entropy theory can be used to capture the correlation of reservoir neurons, assisting the design of better ESNs.

## Figures and Tables

**Table 1:** Complexity measure on time series MG, Lorenz, NARMA and CO.

Indicator	MG	Lorenz	NARMA	CO
PE	0.55409	0.75621	0.97349	0.96773

**Table 2:** Measure on Pearson correlation between $H_{p}^{G}$ and ESN performance in the MG, Lorenz, NARMA and CO prediction tasks, where the change steps of *N* and ρ are 50 and 0.1, respectively.

Step	Parameter Space	Correlation	MG	Lorenz	NARMA	CO
1	N∈[50,500]	$H_{p}^{G}$ vs. MSE	−0.96069	−0.95188	−0.85053	−0.69467
		$H_{p}^{G}$ vs. MC	0.928394	0.971848	0.791282	0.931725
	ρ∈[0.1,1]	$H_{p}^{G}$ vs. MSE	−0.47532	−0.41578	−0.52174	−0.39389
		$H_{p}^{G}$ vs. MC	0.588914	0.189191	0.376211	0.689503
3	N∈[50,500]	$H_{p}^{G}$ vs. MSE	−0.91901	−0.89865	−0.8507	−0.79467
		$H_{p}^{G}$ vs. MC	0.939181	0.921805	0.841936	0.92253
	ρ∈[0.1,1]	$H_{p}^{G}$ vs. MSE	−0.56666	−0.348145	−0.56681	−0.41327
		$H_{p}^{G}$ vs. MC	0.466588	0.27456	0.59542	0.35036
5	N∈[50,500]	$H_{p}^{G}$ vs. MSE	−0.92581	−0.93273	−0.94301	−0.86987
		$H_{p}^{G}$ vs. MC	0.927302	0.920522	0.921338	0.91253
	ρ∈[0.1,1]	$H_{p}^{G}$ vs. MSE	−0.635644	−0.45148	−0.18493	−0.23585
		$H_{p}^{G}$ vs. MC	0.53558	0.554063	0.231826	0.666532

**Table 3:** GSE and MSE of ESN at different scales in the MG, Lorenz, NARMA and CO prediction tasks.

s	MG		Lorenz		NARMA		CO	
	MSE	$H_{p}^{G}$	MSE	$H_{p}^{G}$	MSE	$H_{p}^{G}$	MSE	$H_{p}^{G}$
1	6.33 × 10${}^{-4}$	0.973	3.53 × 10${}^{-4}$	0.968	6.22 × 10${}^{-3}$	0.975	2.73 × 10${}^{-4}$	0.973
2	1.14 × 10${}^{-3}$	0.945	6.59 × 10${}^{-4}$	0.93	6.44 × 10${}^{-3}$	0.945	2.77 × 10${}^{-4}$	0.944
3	1.20 × 10${}^{-3}$	0.933	6.52 × 10${}^{-4}$	0.902	6.68 × 10${}^{-3}$	0.89	2.96 × 10${}^{-4}$	0.906
4	1.26 × 10${}^{-3}$	0.892	7.77 × 10${}^{-4}$	0.901	6.76 × 10${}^{-3}$	0.888	3.04 × 10${}^{-4}$	0.889
5	1.30 × 10${}^{-3}$	0.872	8.82 × 10${}^{-4}$	0.871	6.78 × 10${}^{-3}$	0.863	3.15 × 10${}^{-4}$	0.835
6	1.36 × 10${}^{-3}$	0.827	1.06 × 10${}^{-3}$	0.813	6.79 × 10${}^{-3}$	0.827	3.26 × 10${}^{-4}$	0.83

## Data Availability

The datasets for this study are available upon request from the corresponding author.

## Abbreviations

Abbreviations and notations in the paper:

**Abbreviations**	**Description**
ESN	Echo state network
PE	Permutation entropy
ISE	Instantaneous state entropy
GSE	Global state entropy
RC	Reservoir computing
RP	Recurrence plots
ASE	Average state entropy
SCM	Statistical complexity measure
MCPE	Multiscale complexity permutation entropy
MPE	Multiscale permutation entropy
CECP	Complexity–entropy causal plane
MG	Mackey–Glass
NARMA	Nonlinear autoregressive moving average
CO	Crude oil
MSE	Mean square error
MC	Memory capacity
**Notation**	**Description**
x(t)	The state of RC at time *t*
$x_{n}$	The nth neuron of RC
*W*	Internal weight matrix of ESN
*N*, *n*	The number of RC neurons
*T*, *t*	Time
*X*	Internal state matrix of the reservoir
*Y*	Output matrix of ESN
*m*	Embedding dimension
τ	Delay time
$H_{p}^{t}$	The state entropy of all neurons in the reservoir at time *t*
$H_{p}^{n}$	The time-varying state entropy of nth neuron
$H_{p}^{I}$	The value of ISE
$H_{p}^{G}$	The value of GSE
*s*	The scale factor
ρ	The spectral radius of reservoir
$C_{js}$	The value of SCM
